# Optimising treatment in opioid dependency in primary care: results from a national key stakeholder and expert focus group in Ireland

**DOI:** 10.1186/s12875-018-0792-8

**Published:** 2018-06-30

**Authors:** Marie Claire Van Hout, Des Crowley, Aoife McBride, Ide Delargy

**Affiliations:** 1 Public Health Institute, Liverpool John Moore’s University, Liverpool, UK; 2 Substance Misuse Programme, Irish College of General Practitioners, Dublin, Ireland

**Keywords:** Opioid dependence, Opioid agonist treatment (OAT), Methadone treatment Programme, General practitioner (GP), Ireland

## Abstract

**Background:**

Treatment for opioid dependence in Ireland is provided predominantly by general practitioners (GP) who have undergone additional training in opioid agonist treatment (OAT) and substance misuse. The National Methadone Treatment Programme (MTP) was introduced in 1998, and was designed to treat the opioid dependent population and to regulate the prescribing regimes at the time. The past two decades have seen the increased prescribing of methadone in primary care and changes in type of opioid abused, in particular, the increased use of over the counter (OTC) and prescription medications. Despite the scaling up of OAT in Ireland, drug related deaths however have increased and waiting lists for treatment exist in some areas outside the capital, Dublin. Two previous MTP reviews have made recommendations aimed at improving and scaling up of OAT in Ireland. This study updates these recommendations and is the first time that a group of national experts have engaged in structured research to identify barriers to OAT delivery in Ireland. The aim was to explore the views of national statutory and non-statutory stakeholders and experts on current barriers within the MTP and broader OAT delivery structures in order to inform their future design and implementation.

**Methods:**

A single focus group with a chosen group of national key stakeholders and experts with a broad range of expertise (clinical, addiction and social inclusion management, harm reduction, homelessness, specialist GPs, academics) (*n* = 11) was conducted. The group included national representation from the areas of drug treatment delivery, service design, policy and practice in Ireland.

**Results:**

Four themes emerged from the narrative analysis, and centred on *OAT Choices and Patient Characteristics; Systemic Barriers to Optimal OAT Service Provision; GP Training and Registration in the MTP,* and *Solutions and Models of Good Practice: Using What You Have.*

**Conclusion:**

The study identified a series of improvement strategies which could reduce barriers to access and the stigma associated with OAT, optimise therapeutic choices, enhance interagency care planning within the MTP, utilise the strengths of community pharmacy and nurse prescribers, and recruit and support methadone prescribing GPs in Ireland.

**Electronic supplementary material:**

The online version of this article (10.1186/s12875-018-0792-8) contains supplementary material, which is available to authorized users.

## Background

Opioid dependence is a chronic, relapsing disorder with permanent metabolic deficiency [1], and characteristically complex in terms of patient care, pharmacological, psycho-social and relapse prevention modalities, and treatment outcomes [2, 3]. Ireland currently provides opioid agonist treatment (OAT) to those suffering from opioid dependence within a model of care which acknowledges the central role of the specialist trained general practitioner (GP) in primary care. In Ireland OAT is commenced by suitably trained specialist (GPs) in either addiction clinics or general practice settings (Level 2 GP). Once the patient is stabilised on OAT, referral to Level 1 GPs working in the community for ongoing management can occur. Recent studies in 2013 and 2016 indicate a generally positive attitude of prescribing GPs toward methadone treatment. This was also underpinned by their belief that primary care prescribing of methadone is an essential service to drug users in the community, and one that supports a good relationship between the patient and GP [4, 5]. Prescribing GPs work closely with both statutory (funded and operated by the Health Services Executive, HSE) and non-statutory (part funded by the HSE through a service level agreement, SLA) organisations to optimise OAT delivery. Many of the non-statutory groups provide support and advocacy groups and a number of the larger agencies provide residential detoxification facilities. A number of the non-statutory agencies have a national brief and have been pivotal in the expansion of harm reduction and OAT in Ireland. These groups have also advocated for the decriminalisation of drug use along with the setting up of drug consumption rooms. They play a key role in drug policy and advocate for prompt and easy access to OAT.

In terms of OAT pharmacological options, substitution treatment using methadone is the most common formulation, with buprenorphine-naloxone currently available on a limited named patient basis only. Methadone has been available in Ireland since 1992, and was initially restricted in availability to the capital, Dublin. The *‘Report of the Expert Group on the Establishment of a Protocol for the Prescribing of Methadone’* was conducted in 1993. In 1998, the ‘*Misuse of Drugs (Supervision of Prescription and Supply of Methadone) Regulations’* was set up and has since stipulated regulatory structures for treating opioid dependent patients using methadone. The Methadone Treatment Programme (MTP) protocol designed in 1998 guides OAT treatment delivery in primary care, in terms of protocols for methadone prescribing, guidelines and standards for patient management and care, specialist training requirements for GPs, and protocols for clinical audit [6]. Several reviews of the MTP have been conducted, both internally in 2005 by the *‘Methadone Prescribing Implementation Committee’* itself and externally in 2010 [7]. These reviews recommended improved prescribing and quality of practice in both community and primary care, in order to optimise treatment reach and access across the country, and with support from inter-agency referral pathways. All patients on methadone are listed on the confidential Central Treatment List (CTL) with each patient linked to one specific prescriber and a single dispensing site.

The Irish College of General Practitioners (ICGP) provides the specialist addiction training for GPs who prescribe OAT and plays a central role in the provision and auditing of the MTP. Training consists of an on-line training module in order to qualify for a Level 1 contract. A longer course consisting of workshops, on-line modules and a practice improvement project is required to obtain a Level 2 GP contract. Both Level 1 and Level 2 contracts attract additional remuneration for GPs looking after patients on OAT and ongoing audit of patient care is an essential requirement for maintenance of the contract. Since 1998, the number of prescribing GPs has risen steadily each year and there are currently (mid 2017), a total of 345 Level 1 GPs and 57 Level 2 GPs providing OAT treatment in primary care.

Since the introduction of the MTP greater prescribing of methadone in primary care is observed (Central Treatment List). As mentioned, during the early years of the MTP, heroin use and treatment were mainly confined to the capital, Dublin. In more recent times, the opioid misuse problem has spread to outside the capital, and regional OAT structures have struggled to meet the demand resulting in waiting lists in areas outside of Dublin. There have also been increasing drug related deaths and changes in the type of opioid abused (over the counter and prescription medications). Given that GPs currently provide the clear majority of OAT in Ireland across a variety of settings, the ICGP conducted a focus group study to investigate national stakeholder views around current provision of the MTP, barriers experienced and perspectives around how to improve its design and implementation in Ireland.

## Methods

### Aim

The aim was to explore the views of national statutory and non-statutory stakeholders and experts on current barriers within the MTP and broader OAT framework in order to inform their future design and implementation.

### Approach

A qualitative study using a single focus group with a purposive sample of national key stakeholders and experts, with a broad range of expertise (clinical, addiction and social inclusion management, harm reduction, homelessness, specialist GPs, academics) was conducted. The research team selected participants to ensure national representation. Seven of the eleven experts have a national brief to their roles and oversee OAT design and implementation across the entire country. Eight of the eleven participants participate at a national level in drug related policy. The participants were also selected to ensure that non-statutory agencies were adequately represented and that these groups had a national brief (*n* = 3). The focus group was conducted in Dublin to facilitate the largest number of participants but teleconferencing facilities were made available to those unable to travel (n = 3). A focus group guide using four broad questions (see Additional file 1) was designed by the team, which consisted of the Director and Assistant Director of the Substance Misuse Programme (SMP) at the ICGP, the Clinical Audit Facilitator (CAF), who is also an academic, and the administrator of the SMP. The guide explored the identification of patient, system and clinical barriers and enablers to accessing and engaging with OAT, immediate and long term solutions to enhancing OAT provisions in the community, and models of good practice and lessons learnt which could be shared nationally and incorporated into the revised MTP.

### Ethical and study procedure

Ethical approval was granted by the ICGP. Chosen stakeholders were sent an email with information around the focus group aims and objectives, procedures around anonymity and voluntary withdrawal assurances, and with an invitation to attend the focus group. The focus group took place at the ICGP premises in the Irish capital, Dublin. For non-attenders (*n* = 2) teleconferencing facilities were made available All participants signed a consent form permitting audio-recording. The focus group was facilitated jointly by author one and author four. Following the focus group, the audio recording was transcribed and destroyed. All data in the transcript was anonymised.

### Data analysis

A content analysis of the data was undertaken by author one and two, which involved open, axial and selective coding resulting in the generation of listing of key concepts, ideas, words and phrases, formulating main and sub categories, and generating overarching themes.

## Results

Both statutory (ST) and non-statutory agencies (NST) with a gender balance were represented (*n* = 11). Six males and five females participated in the focus group, with four specialist GPs, four ST (funded and operated by the HSE) and three NST stakeholders (part funded by the HSE through an SLA) represented. Four themes emerged from the analysis of narratives, and are presented here with illustrative quotes.

### OAT choices and patient characteristics

Initial discussions centred on the stigma toward OAT in Ireland, and the general public and drug users’ negative attitude towards it. Comments were made around the lack of choice in OAT in Ireland, with methadone available nationally for the majority of patients and buprenorphine-naloxone (trade name Suboxone) restricted to specific patient cohorts. In contrast to the stigma attached to methadone, patients appeared to have more favourable attitudes with regard to Suboxone, which is seen as a medical treatment.

*‘We need to have Buprenorphine available through the pharmacies nationally and not to prohibit its use.’ [SpGP1]*

Additional changing patterns in opioid drug abuse were observed by the group, with a shift toward increased dependence on prescription and over the counter opioid based analgesics**.** These changing OAT patient characteristics in terms of those with presenting with prescription and OTC opioid abuse (as opposed to heroin), and the difficulties for such patients given the stigma and location in accessing mainstream addiction clinics which generally treat heroin addiction were discussed and central to the requirement to expand choice in OAT.

*‘Patterns are changing, over the counter painkillers, reduction in heroin users but our models of treatment haven’t changed accordingly.’ [SpGP1]*

Concerns were voiced around issue of OAT patient co-dependence on other substances generally alcohol, and benzodiazepines and Z-hypnotics, both prescribed and sourced on the street. Participants described difficulties in management of these poly dependencies. National assessment, referral and detoxification pathways for benzodiazepine and Z-hypnotic drug abuse and dependence were described as lacking. Efforts to manage the problem centred on some service providers refusing to prescribe these drugs to their methadone patients. Patients were described as circumventing this by accessing a GP other than their methadone prescriber.

*‘One of the things that puts GPs off even though it is not directly related to methadone it’s a whole big mess of benzo and tablet problems.’ [SpGP3]*

Behavioural issues due to poly substance intoxication was also viewed as problematic for primary care and community pharmacy staff who dispense methadone, and at times requiring security measures.

*‘There is a problem particularly for pharmacies as well as GPs…pharmacies are a business and they can’t afford to have someone coming in and causing chaos in a pharmacy’[SpGP2]*

Some problems are evident with regard to all female GP practices and the supervision of drug screening for male methadone patients.

*‘We have no men in our practice at the moment. So supervising men is a problem for us’. [SpGP3]*

Long term methadone patients along with the aging methadone patient population were viewed as creating a draw on services. Discussions centred on the adaptation of service models given the aging population of both drug users and methadone patients.

*‘We have to recognise it is an ageing model and in Dublin…I think we need to be very careful about setting up new models that are potentially very expensive for a profile that may not exist in 10 or 15 years time.’ [SpGP1]*

Participants described the complexities of treating and engaging with homeless drug users, and the difficulties around long term methadone treatment. In terms of attempting to reduce patients and taper off methadone, participants described the need for a broader de-medicalised approach to recovery. Debate occurred with regard to the Irish stipulation for opioid free urines prior to accessing a detoxification centre.

*‘People have to have 3 or 4 urines that are opioid-free before they can be admitted to a centre…if they are able to manage 3 or 4 urines that are opioid-free then they don’t need to go into the detox centre in the first place...’ [NS2]*

### Systemic barriers to optimal OAT service provision

National provision of OAT and dispensing of methadone was described as patchy, and largely concentrated in the capital, Dublin, and larger urban areas. Some participants voiced concern around the need for more Health Service Executive dispensing centres as a way of dealing with national demand, particularly in the context of destabilisation of patients and the current requirement to resume initiation of treatment in the clinics. Other logistical complexities for patients centred on lack of rural GPs and community pharmacies willing to prescribe and dispense methadone, rural residences and cost of transport, particularly outside of the capital. As outlined in the previous theme, stigma of methadone, and the lack of choice with large methadone clinics in some areas offering the only route to treatment were viewed as representing fundamental systemic barriers to OAT access. Service level barriers to access for individuals experiencing opioid dependence were described as centring on the complexities around the patients address of residence with regard to options to access stabilisation OAT in clinics or by a Level 2 GP, their general preferences to attend primary care for OAT, and lack of availability of Level 2 GPs in the community. Many participants described long waiting lists and under capacity of local services to deal with the issue of opioid dependence, and provide the current requirement for regularity of consultations.

*‘There is a problem with waiting lists and I think nationally there needs to be a more robust, systematic review of waiting lists and if a patient is waiting for more than 3 months for treatment there needs to be a proper analysis*’*. [SpGP1]*

*‘If there were more Methadone prescribers within the GP community then there would be no need for these people there in the country to travel to access treatment’. [NS2]*

Other blocks centred on homeless patients seeking treatment with no fixed address, and the treatment influx from parts of the country outside the capital.

*‘Where are the homeless people going? This is not a good model of care. Having them sent to multiple pharmacies and multiple centres causes violence and antisocial behaviour, and in fact you are creating more problems and the treatment is bringing problems with it.’ [SpGP1]*

The MTP given its stipulation to stabilise patients in addiction clinics or by Level 2 GPs prior to referral to the community Level 1 GP was viewed as not operating efficiently. The restriction of numbers of patients managed by Level 1 GP (*n* = 15) in the community was central to this issue and was viewed as contributing to long waiting lists.

*‘Information we are getting is that everybody and everything has to go through the clinic…we have Level 2 GPs… I would be saying why are we not utilising the L2 GPs to the max and not be creating waiting lists.’ [SpGP4]*

### GP training and registration in the MTP

Participants discussed the specialist Level 1 and 2 training and Health Service Executive registration complexities as systemic barriers to providing optimal OAT in Ireland. Stigma of OAT within medical practice and education was viewed as affecting training uptake. Those involved in GP training (and who prescribe methadone) described the willingness of younger doctors to engage in training when exposed to OAT, and particularly when hosted by larger GP practices involved in methadone prescribing. GP registrars not exposed to the opioid dependent patient cohort were described as not willing, and similar was described with regard to newly qualified pharmacists.

*‘You would like to think that GP trainers would be the frontline for educating people being open to the idea that all patients are equal…… the majority of GP trainers that we have do not do methadone and would not entertain methadone treatment. There are messages like that going out to trainees’ [SpGP4]*

Difficulties centred on the lack of uniform approach to mentoring younger GPs, and the current requirement for methadone contracts to be assigned to a practice address, not the prescriber. The Level 1 and Level 2 structures were viewed as complex and difficult, particularly for newly qualified GPs entering employment and securing employment in primary care practices not part of the MTP.

*‘It is an incredible missed opportunity, every GP trainee in the country should be obliged to do Methadone training like they are obliged to do the Women’s Health. [SpGP3*]

Another systemic issue in the MTP was described as centring on the significant effort, organisation and commitment in the contractual difficulties to become and register as a Level 2 prescriber which was viewed as deterring some Level 1 GPs from progressing.

*‘Its too difficult to get to a Level 2 scenario…if you have done the Level 1 training, to get to a Level 2 prescriber is too difficult. It’s a long process.’ [SpGP2]*

Complexities of the GPs role in supporting the opioid dependent patient were discussed in terms of length of patient consultation, the myriad of additional health conditions and social challenges. In some areas Level 1 GPs were under resourced despite the funding allocation for OAT patients, and unable and not willing to take on more complex patients.

*‘Methadone is well remunerated... I don’t begrudge any of our methadone users the time they take up. But new GPs won’t start because it’s so complicated,’*[*SpGP3]*

### Solutions and models of good practice: Using what you have

Firstly, participants discussed potential solutions and best practices for shared learning. Several key areas were identified, with first centring on the requirement for all GP registrars to be trained in methadone prescribing and the treatment of opioid dependence and related health problems. The ICGP has long held the view that all GPs should be in a position to provide methadone and other opioid agonist treatment in primary care ‘*to be part of routine GP primary care’*. Encouraging GPs to change attitudes, and engage in the specialist training via mentoring of more experienced GPs was discussed, and appeared to represent a way of reducing fears and concerns around engaging with the methadone patient cohort.

*‘I’d like to see that Level 2 would become more specialised and that Level 1 would almost become normal for GPs so that they have facilities for benzos and for other addictions.’[SpGP2]*

Secondly, the group discussed how to optimise the available resources within the current MTP. Finding ways for supporting OAT patients via shared care planning with available community agencies was viewed as vital within the MTP. Addiction clinics were viewed as having a range of supports available to patients. Avenues for potential support for community practitioners centred on the available outreach, social, community and psychological support services, and engaging with case workers from local Drug Task Forces.

*‘There is a perception among GPs like me who are doing methadone versus the clinics is that the clinics have a lot of services that we don’t get so easily, like the counselling services, …if you were able to offer GPs some of those supports…once a month or something like that, that would be just as good as having a full blown clinic’. [SpGP3]*

Informal meetings between staff were viewed as important to help share issues and support each other within the practice, particularly if GPs were working part time.

*‘The work is too complex to be able to manage it on your own’. [SpGP3]*

*‘We tend to be the key worker, because we are the only person that these people are seeing.’[SpGP1]*

Using family support systems where possible from treatment onset was also viewed as a potential lesson learnt. Complexities arise when patients have no family or are homeless. Shared care and key working was viewed as very important.

‘*Resources out there that are probably underutilized at the moment…for example, voluntary based services around the corner from the GP. It is about getting to know the person. It is about case management in all areas of their life.’ [NS1]*

Thirdly, given the logistical barriers for patients in rural areas, or areas with no Level 2 GP, the group discussed the potentials for utilising community pharmacy and nurse prescribing in the community. Complexities centred on this recommendation, and current service level agreements.

*‘I would see a lot of what’s done by the doctor, could be done by the nurses…and the doctor then can be able to prescribe more …and be able to look after more in terms of the monitoring, the supervision, the diagnosis of mental illness.’[SpGP1]*

Lastly, the remit of community pharmacy could expand to support work in primary care in terms of extended dispensing, education and vaccination of drug users. Community pharmacies could expand to take on the role of patient vaccination (Hepatitis A and B) within their role in providing needle and syringe exchange.

*‘Another job that pharmacists might take on is Hepatitis A & B vaccination in pharmacies. It’s not an immediately practical thing but something definitely to think of in the future’. [SpGP2]*

*‘Down the country, why not augment the community pharmacies with extra staff. The 7 day pharmacies that are open.’[SpGP1]*

## Discussion

The study illustrates the complexities around the MTP within primary care in Ireland, along with the systemic failures in optimal service provision for opioid dependent individuals, and challenges encountered in managing opioid drug users. Primary care providers can take a proactive role in treatment of opioid dependence [8–10] and so enhance health care provision [11, 12]. Integration of OAT into primary care via different models can expand access to treatment [13]. Mainstreaming of OAT into primary care can also help to reduce stigma as a barrier to treatment uptake [14, 15]. Systemic barriers observed by these national stakeholders and experts in Ireland were similar to those reported elsewhere and centre on stigma, lack of therapeutic choice in Ireland, reluctance of GPs to prescribe OAT, and complex reimbursement systems [16–21]. Lack of MTP coverage across the country was illustrated and represents a systemic barrier to access for patients living in rural areas, homeless patients without a residential address, and those seeking treatment due to long waiting lists. Similar issues have been reported in other countries exploring the expansion of OAT into community and primary care [13, 21, 22]. The expansion of buprenorphine-naloxone availability could overcome this barrier. In many jurisdictions buprenorphine-naloxone availability in primary care has allowed for the rapid expansion of OAT. Buprenorphine’s use as a combined product with naloxone has allowed for a safe reduction in supervision requirements and increased utility in patients living in isolated areas with poor access to medical and pharmacy services. The use of tele-medicine linking less experienced rural GPs with their more specialist colleagues could further increase OAT coverage nationally.

Participants described the complexities of the current Irish opioid dependent population in terms of long term and aging patients, co-dependencies on other drugs such as benzodiazepines and Z-hypnotics, abuse of prescription and OTC opioid analgesics, and homelessness. These complexities of opioid dependent patients in terms of psychiatric co-morbidity, and co- dependencies are well evidenced in the literature [10]. Similar to other countries, primary care practice based pressures centre on patient behavioural issues and resources required to support longer consultation times due to the health and social care challenges of these patients. Studies have reported on GP reluctance to prescribe methadone due to their fears around patient behavioural issues, the complexities of opioid dependent patients, concerns around workload and the time required to manage such patients, and staff safety [4, 6, 16, 17, 23–29]. Van Hout and Bingham [4] have underscored the multiplicity of roles (patient advocate, medical supervisor and detoxification gate keeper) that GPs have when involved in prescribing methadone.

Strategies to address systemic barriers centre on the expansion of training, increased use of community pharmacists, development of the nurse prescribing role and promoting the easy access to GPs via key working [13]. Shared care with available community based services was viewed as vital in terms of family support, key working, outreach and psycho-social support. The lack of therapeutic choice in Ireland needs to be addressed. Buprenorphine is underutilised in Ireland due its restricted availability, but has been reported as safe and effective in OAT in primary care [21]. Providing this OAT option could lessen the draw on resources and support OAT patients across the country. Other potential solutions using the available resources in the MTP centred on expanding the remit of the community pharmacy in terms of patient education and vaccination, and the role of the nurse prescriber. Nurse prescribers can overcome systemic barriers and failures and improve access to OAT [21]. Technology using E-consultation and e-prescribing to support patients who have to travel long distances for treatment could also be considered and would facilitate access to Level 2 GP services.

Similar to research in the United States [10] and building on the primary care model now widely accepted in Europe, mainstreaming of OAT has many advantages, and success will depend on service delivery models and the improved and expanding training of doctors in Ireland. GPs are ideally placed to diagnose patients with substance related problems and require a specific skill set to provide clinical care. The focus group highlighted the need to ensure newly qualified GPs are trained in OAT and to support those interested in securing Level 1 and Level 2 contracts. Participants echoed views reported by the ICGP in 2016, where a need for continued support of prescribing GPs (Level 1 and 2), training of new GPs and encouragement of further specialisation to Level 2 were identified [5]. Issues around encouraging newly qualified GPs to engage in provision of the MTP service were described, and support research reporting on newly qualified GPs having a more positive attitude toward opioid dependent patients and self-awareness of competencies to treat this condition [30, 31]. Training at undergraduate and registrar levels is warranted [10]. No Irish medical school has any elective or integrated training in addictions, and with no documented drug and alcohol teaching sessions [32, 33]. Particularly in undergraduate training, addiction as a disease should be integrated into pre-clinical course material, and careful emphasis on development of positive attitudes to working with addicted patients is warranted [34–36]. Hussein Rassool [37] has indicated that substance misuse training can contribute to an increase in confidence in participants in working with substance misusing patients. Research elsewhere has underscored the need to integrate addiction medicine into medical and primary care registrar education, given the public health cost of medical, behavioural and social problems associated with substance use, and also given the frequent lack of recognition of substance abuse and failure to provide appropriate treatment on the part of general practitioners [38–43].

The use of the focus group methodology in this study allowed for the efficient collection of the views of a very diverse group of Irish addiction experts in relation to the blocks and facilitators to OAT in Ireland. The inclusion of both statutory and non-statutory experts allowed for robust and insightful discussion and the focus group methodology is recognised as a good research method to capture the richness of these discussions. The inclusion of experts in the area of policy development and implementation along with experts in treatment design and delivery allowed for an in-depth exploration of the issues.

There are a number of limitations to this study. The findings are limited to the data collected from only one focus group which contained only 11 experts. While the research team endeavoured to ensure national representation it is reasonable to assume that this group is not fully representative of all regions and there are deficits in recognising all the barriers and enablers to OAT in Ireland. The focus group did not include patients or patient representatives. A further limitation is that the focus group was conducted by, or included, those who have responsibility for the SMP. The researchers recognised this and attempted to limit this conflict of interest by picking researcher 1 as the group facilitator. This researcher would have had the least prior involvement with the focus group participants. Lastly, the involvement of the members of the SMP in the focus group may have impacted on participants’ willingness to share their views fully for fear of antagonising or upsetting these SMP members.

## Conclusion

The study is a first step in a process to identify barriers to optimal OAT provision by GPs in Ireland. It has successfully identified a number of previously unrecognised issues that will be progressed through a number of national drug treatment and policy groups. Key national stakeholders and experts identified a series of improvement strategies which can reduce OAT stigma and barriers to access, optimise therapeutic choice, enhance interagency care planning within the MTP, utilise the strengths of community pharmacy and nurse prescribers, and recruit and support methadone prescribing GPs. The ICGP will advance the implementation of these recommendations through a number of national drug treatment and policy groups and will plan and undertake a series of independently run expert focus groups across the country to gain further insight into this topic and add to these recommendations.

## Additional file

Additional file 1: Focus group guide. Questions used in facilitation of the focus group. (DOCX 12 kb)

## Abbreviations

CAF: Clinical Audit Facilitator
CTL: Central Treatment List
GP: General Practitioner
HSE: Health Service Executive
ICGP: Irish College of General Practitioners
MTP: Methadone Treatment Programme
NST: Non Statutory Stakeholder
OAT: Opiate Agonist Treatment
SLA: Service Level Agreement
SMP: Substance Misuse Programme
ST: Statutory Stakeholder
