# Supplementary material for: Optimising treatment in opioid dependency in primary care: results from a national key stakeholder and expert focus group in Ireland
Source: BMC Fam Pract. 2018 Jun 30;19:103. doi: 10.1186/s12875-018-0792-8 (PMC6026515; doi:10.1186/s12875-018-0792-8)
Supplement: Supplementary file 1 — Focus group guide. Questions used in facilitation of the focus group. (DOCX 12 kb) [file 12875_2018_792_MOESM1_ESM.docx]

**Focus Group Guide**

Can you describe your views around current provision of the MTP?

Can you describe any current patient, system and clinical barriers within the MTP?

Can you describe any immediate and long term solutions to enhancing OAT provisions in the community

Can you describe models of good practice and lessons learnt which could be shared nationally and incorporated into the revised MTP?
